# Supplementary material for: mTOR Inhibition Impairs the Activation and Function of Belatacept-Resistant CD4+CD57+ T Cells In Vivo and In Vitro
Source: Pharmaceutics. 2023 Apr 20;15(4):1299. doi: 10.3390/pharmaceutics15041299 (PMC10142381; doi:10.3390/pharmaceutics15041299)
Supplement: Supplementary file 1 [file pharmaceutics-15-01299-s001.zip › pharmaceutics-2227425-supplementary.pdf]

Supplementary figure

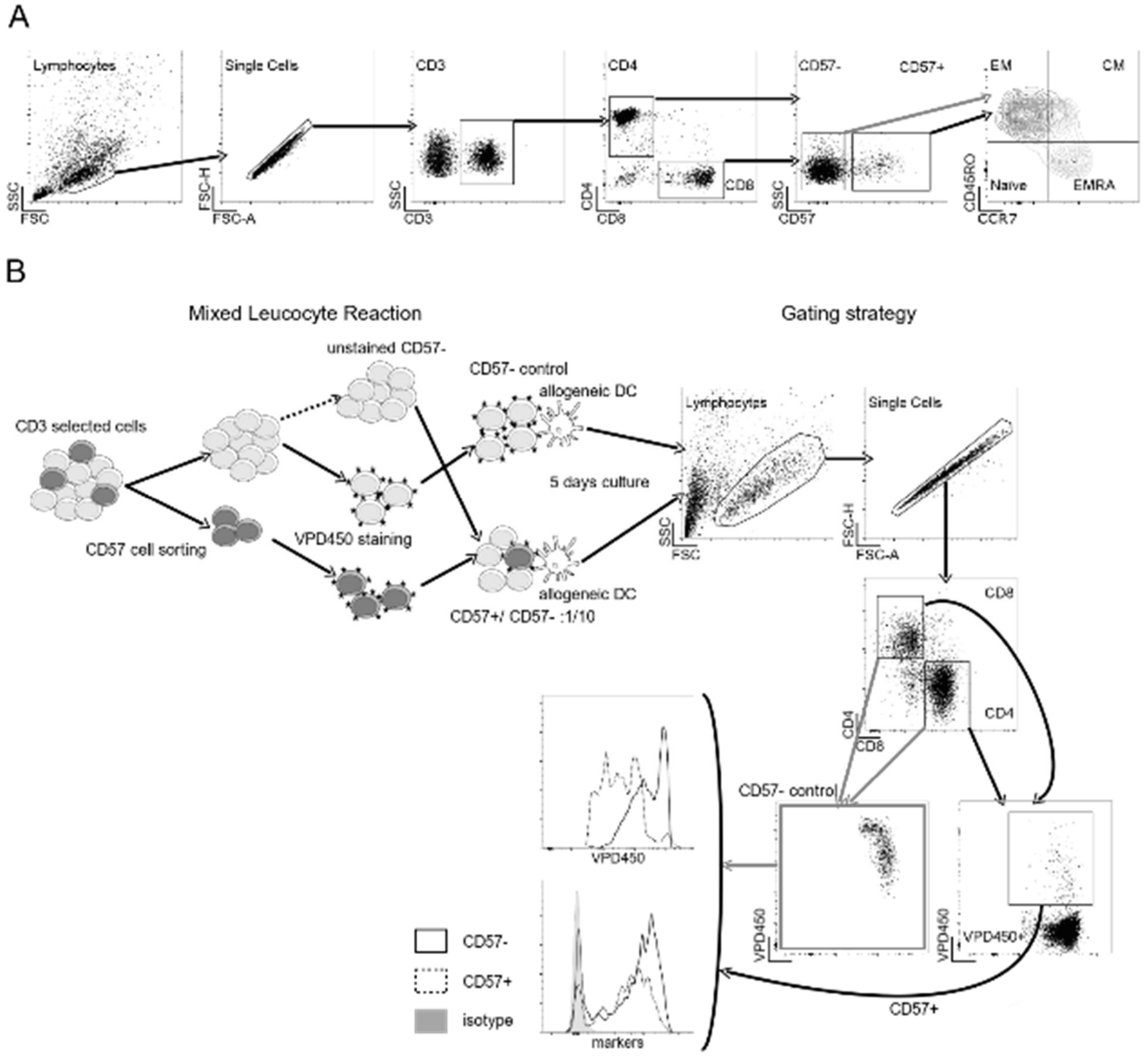

Figure S1. Gating strategies and MLR culture protocol diagram. (**A**) Gating strategy used to define memory T cells populations. (**B**) MLR culture protocol and gating strategy is depicted.
